# Solution‐Processed Diode‐Like ZnO Nanoparticle Device with Tunable Threshold Voltage and Super‐Nernstian Ion Sensitivity

**DOI:** 10.1002/smll.202504332

**Published:** 2025-06-12

**Authors:** Mengyang Qu, Huanghao Dai, Omesh R. Kapur, Stephen P. Beeby, Harold M. H. Chong

**Affiliations:** ^1^ School of Electronics and Computer Science University of Southampton Southampton SO17 1BJ UK

**Keywords:** ionized molecule, ion adsorption and migration, nanoparticles, pH sensor, Zinc Oxide

## Abstract

This study introduces a fabrication method to produce a zinc oxide nanoparticles (ZnO NPs) diode‐like interface device for sensing applications. This structure is achieved via the modulation of ionized oxygen molecules adsorbed on the surfaces of the ZnO NPs, distinguishing it from the conventional diode devices. The device exhibits an on/off ratio of 10^5^ and features a tunable threshold voltage contingent upon varying surface charge conditions, positioning it as a promising candidate for high‐sensitivity chip‐level pH sensing applications. A highly sensitive pH sensor based on this interface is successfully fabricated using a fully solution‐based process, excluding any high‐temperature steps. As the pH value of the test solution decreases, the sensor demonstrates an increase in threshold voltage, achieving a super‐Nernstian sensitivity of 360 ± 11 mV pH^−1^. The fabrication process reaches a maximum temperature of 120 °C and employs a UV‐vacuum‐heating (UVVH) technique. To maintain the electrical integrity of ZnO NPs, ethylene‐vinyl alcohol (EVOH) is utilized to provide a protective, waterproof, and oxygen‐barrier passivation layer. The operational behavior and diode‐like characteristics of the sensor, attributes to ionized oxygen molecule adsorption on ZnO NPs, are accurately predicted using a combined adsorption isotherm and electrical model, aligning well with experimental results.

## Graphical abstract

The working function of ZnO NPs diode‐like devices and their ion sensing applications. This structure is achieved by modulating the ionized adsorbed oxygen molecules on the ZnO NPs surface. The adsorption and desorption can be controlled by the external electrical field to switch on and off. This behaviour can be affected the surface charges positioning it as a high sensitivity pH sensor.

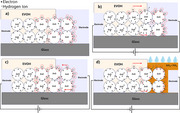

## 1 Introduction

Zinc oxide (ZnO) stands out among metal oxides as a low‐toxicity, sustainable semiconductor with diverse electronic applications, including transistors.^[^ ^1^, ^2^ ^]^ and memristors.^[^ ^3^, ^4^, ^5^ ^]^ Its wide bandgap, thermal stability, and high carrier mobility contribute to its suitability for these devices.^[^ ^6^, ^7^ ^]^ There are various techniques for the deposition of ZnO, including but not limited to atomic layer deposition (ALD),^[^ ^8^ ^]^ sputtering,^[^ ^9^ ^]^ spin‐coating,^[^ ^10^, ^11^ ^]^ and printing.^[^ ^12^ ^]^ Nonetheless, achieving ZnO films with superior properties often requires relatively high temperatures greater than 300 °C^[^ ^13^ ^]^ or stringent fabrication conditions such as vapor deposition.^[^ ^14^ ^]^ Among the various methods employed, solution‐processed technology is recognized as a cost‐effective and efficient deposition technique. In this approach, the target substance is dissolved or dispersed in an appropriate solvent and subsequently deposited onto the selected substrate using printing or spin coating methods. In contrast to conventional vapor deposition techniques, the solution‐processed deposition of ZnO nanoparticles (NPs) does not impose stringent requirements regarding the deposition environment or energy consumption. While solution‐processed ZnO NPs are straightforward and compatible with diverse substrates, the resulting films generally exhibit high resistivity and low electrical stability.^[^ ^10^ ^]^

According to our previous studies,^[^ ^10^ ^]^ oxygen molecules may adsorbed on the surface of ZnO NPs and ionized in the atmospheric environment, which will greatly increase the resistivity of ZnO NPs. Environmental factors such as light and humidity can influence the interaction between ZnO NPs and ionized oxygen molecules, potentially resulting in instability of the electrical properties. The UV‐vacuum‐heating (UVVH) process has been shown to reduce the resistivity of ZnO NPs significantly. Furthermore, passivation using ethylene‐vinyl alcohol (EVOH) polymer is found to enhance the electrical stability of these nanoparticles substantially. By varying the types and thicknesses of the passivation layers applied, the adsorption process can be tailored, thereby influencing ZnO NPs resistivity through the substances adsorbed on their surfaces. The study indicates that in the absence of a passivation layer, ZnO NPs exhibit a notable increase in resistivity, with a dramatic escalation (by a factor of 10^6^) observed one day post‐UVVH treatment compared to the immediate post‐treatment measurement. In contrast, ZnO NPs films with a thin EVOH passivation layer showed a gradual increase in resistivity. As the thickness of the passivation layer was augmented, the rate of resistivity changes diminished. Significantly, an 80 µm EVOH passivation layer maintained stable resistivity after 60 days of storage in dark ambient environment. These findings suggest that the resistivity of ZnO NPs can be modulated by controlling the amount of adsorbed ionized oxygen molecules on their surfaces.

The operating principle of metal oxide ion or pH sensors is based on the ability of hydroxyl groups on the metal oxide surface to function as adsorption sites for anions and cations, facilitating ion adsorption and generating surface potential. Different ions produce distinct potentials; specifically, the adsorption of hydrogen ions results in the formation of positive charges, whereas the adsorption of hydroxide ions leads to the development of negative charges.^[^ ^15^ ^]^ Nonetheless, the sensitivity of traditional pH sensors is limited by the Nernst limit (59 mV pH^−1^), which can restrict diagnostic efficacy. Consequently, overcoming the Nernst limit to develop high‐sensitivity pH sensors has garnered considerable research interest. The highest reported sensitivity is 362 mV pH^−1^ which is over six times the Nernst limit.^[^ ^16^ ^]^

The pH value of human body fluids serves as a crucial biological indicator with significant implications for clinical diagnosis. The normal pH range of human blood is between 7.3 and 7.5. A pH level below 7.3 is indicative of acidosis, while a pH level above 7.5 suggests alkalosis.^[^ ^17^ ^]^ In the context of cancer diagnosis, the extracellular environment of cancerous cells exhibits a slightly lower pH than normal cells due to its acidic nature.^[^ ^18^ ^]^ Compared with conventional glass electrodes, metal oxide pH sensors have the characteristics of fast response, high thermal stability, low cost, high integration, and miniaturization. In the field of pH sensors, their high surface area‐to‐volume ratio enhances ion adsorption from the solution, thereby improving sensitivity.^[^ ^19^ ^]^ However, metal oxides are easily affected by environmental factors, such as light, which can cause their electrical properties to be unstable, thus affecting the detection accuracy.^[^ ^19^ ^]^ Recent advancements have focused on developing pH sensors that utilize metal oxides, such as ZnO,^[^ ^20^ ^]^ RuO_2_,^[^ ^21^ ^]^ WO_3_,^[^ ^22^ ^]^ and IrO_2_^[^ ^23^ ^]^ as sensing layers.

Furthermore, nanostructured ZnO showing high surface‐to‐volume ratio and semiconductor properties is promising material for sensing applications,^[^ ^24^ ^]^ such as gas sensors.^[^ ^25^, ^26^, ^27^ ^]^ While ZnO has been explored for pH sensing in medical diagnostics,^[^ ^9^, ^20^, ^24^, ^28^ ^]^ the Nernst limit affects its sensitivity.^[^ ^9^, ^20^ ^]^ However, studies have demonstrated that this limit can be overcome using field‐effect transistors^[^ ^29^ ^]^ or charge‐coupled devices,^[^ ^30^ ^]^ though these introduce complexity in fabrication processes, leading to challenges in manufacturing and ensuring consistent detector sensitivity. To address this, researchers have investigated using nanoparticles (NPs) to increase active surface sites in pH sensors, enhancing device sensitivity.^[^ ^28^ ^]^ In addition, nanoparticle‐based ZnO thin films present a promising avenue due to their ease of synthesis, safety profile, and cost‐effectiveness.

The polarized nature of these adsorbed ionized oxygen molecules suggests that their adsorption and desorption can be effectively manipulated by an electric field.^[^ ^31^ ^]^ It is hypothesized that the resistivity of ZnO nanoparticles can be modulated by the application of an electric field, and the surface charge of the nanoparticles can influence the movement of ionized oxygen molecules. The UVVH process effectively removes ionized oxygen molecules adsorbed on the surface of ZnO, exposing a more significant number of adsorption sites, which increases the operational efficiency of ZnO NPs pH sensors. In this work, a low‐resistance high‐resistance (LR‐HR) ZnO NPs interface was designed and fabricated using UVVH process to achieve substantial control over the device resistance via an externally applied electric field. Using this approach, a novel pH sensor was fabricated by an all‐solution‐processed deposition method, achieving performance beyond the Nernst limit. The device demonstrates diode‐like electrical behavior, with its threshold voltage exhibiting a dependency on the pH of the tested solution. Specifically, the threshold voltage increases from 0.47 to 2.31 V as the pH value decreases from 9 to 4. The sensitivity is measured at 360 ± 11 mV pH^−1^, matching the state‐of‐the‐art, and the ON/OFF current ratio is 10^5^. The operating principle of the pH sensor is also analyzed, and a mathematical model is presented to fit its electrical behaviour to different pH hydrogen ion concentrations.

## 2 Results and Discussion

### 2.1 Fabrication Process

**Figure 1:**
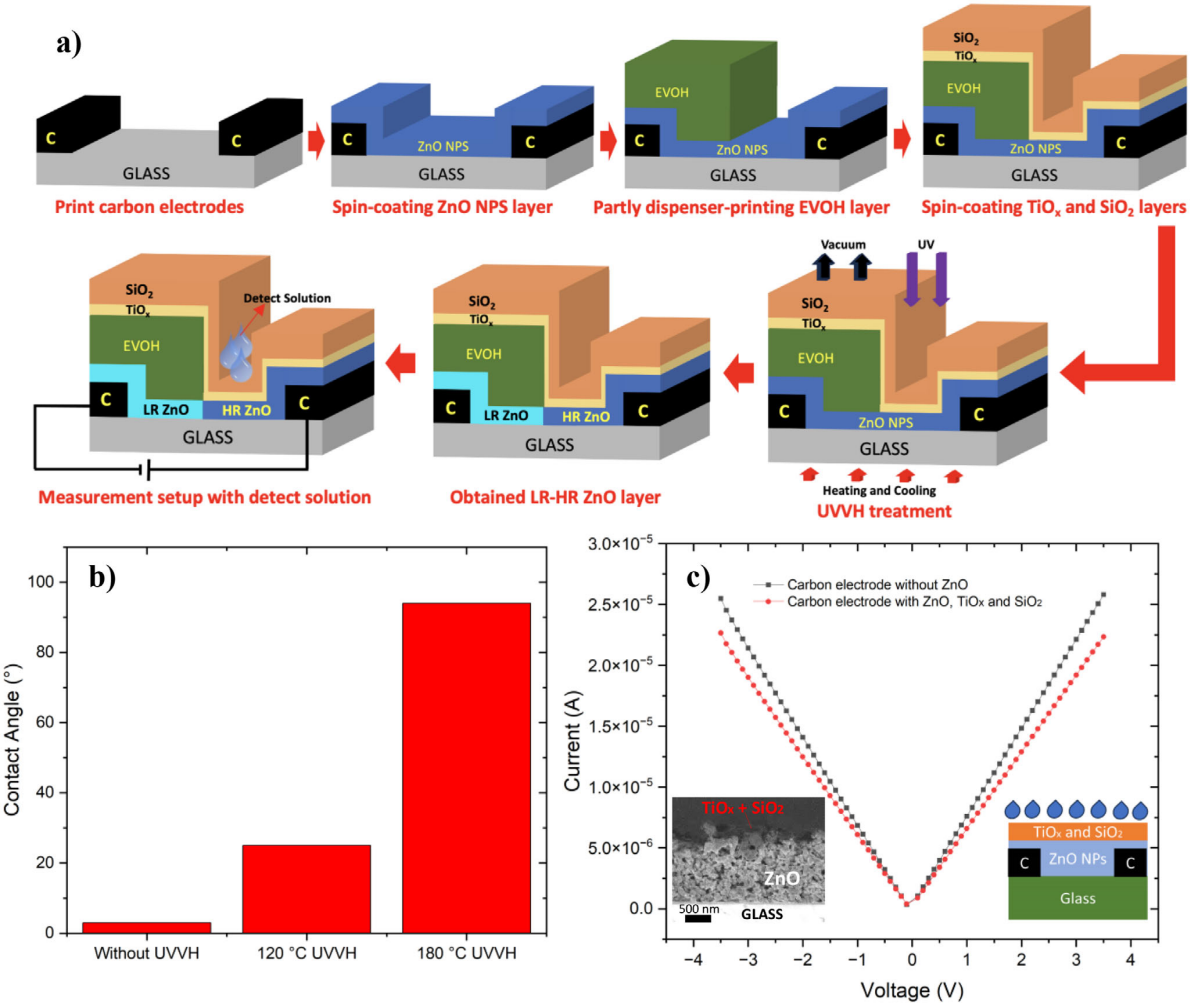
a) The fabrication process of UVVH‐treated ZnO NPs pH sensor. It begins with printing carbon electrodes and spinning the ZnO NPs onto the glass substrate. Dispensing prints the EVOH/DMSO solution on one side of the ZnO NPs and warms it to 80 °C. Step three shows the spinning coats of titanium oxide precursor and silica as the protection for ZnO NPs. Followed by UVVH treatment, heats for 2 h and cools to room temperature. The region for the pH solution is the ZnO NPs that do not contain EVOH. Step five shows the measurement setup. The fabrication process for the intrinsic diode‐like device is shown in the supporting material (Figure S4, Supporting Information), b) Contact angle measurements for ZnO NPs film with different UVVH temperatures. c) I‐V measurement of the DI water used for preparing the pH solution on a device with carbon electrodes without ZnO and a device containing carbon electrodes with the ZnO NPs and TiO_x_ and SiO_2_ layers. Insert shows the SEM cross‐section of ZnO NPs with TiO_x_ and SiO_2_ layers. The film reveals a porous structure that permits water permeation into the ZnO NPs layer.

**Figure** **1** illustrates the fabrication process of the ZnO NPs‐based pH sensor. The titanium oxide and silica particle film, almost insoluble in weak acids and alkali solutions, protected the ZnO NPs while testing and cleaning pH solutions. After deposition, the samples were subjected to UVVH treatment at 120 °C for 2 h, then cooled to room temperature under vacuum and UV conditions. During the UVVH process, the vacuum pump was kept turned on. The detection solution was applied to the ZnO NPs connected to the positive electrode. As previously reported,^[^ ^10^ ^]^ the UVVH process removes surface‐adsorbed ionized oxygen, thereby reducing the resistivity of the ZnO NPs film. The EVOH layer prevents the re‐adsorption of oxygen, maintaining the low resistivity state (LR ZnO). Without EVOH passivation, ZnO NPs rapidly adsorb atmospheric oxygen molecules, leading to a high resistivity state (HR ZnO). Figure S1 (Supporting Information) describes the UVVH‐treated ZnO NPs pH sensor.

The contact angle measurement was shown in the supporting material. Figure S2 (Supporting Information) depicts the contact angle measurements for ZnO NPs across varying conditions and the results were summarised in Figure 1b. Specifically, Figure S2a (Supporting Information) illustrates the contact angle of the as‐deposited ZnO NPs film which is smaller than 10°, while Figure S2b,c presents the contact angles for ZnO NPs films following UVVH treatment at 120 and 180 °C, respectively. Post‐UVVH treatment, the contact angle increased to 25° and 94° respectively, indicating a significant reduction in the surface energy of the ZnO NPs. This reduction is attributed to the removal of the chemical functional groups, such as adsorbed oxygen molecules or hydroxyl groups, from the ZnO NPs surface during the UVVH process. A higher surface energy is vital for solution sensing, as it enhances the adsorption of target substances onto the ZnO NPs film, thereby inducing changes in electrical properties. So, UVVH with 120 °C process was chosen to fabricate the device.

During the UVVH process, ionized oxygen adsorbed onto the surface of ZnO NPs is removed, forming low‐resistivity ZnO (LR ZnO). The subsequent application of EVOH passivation preserves this low resistivity level by hindering the re‐adsorption of oxygen molecules from the ambient atmosphere. In contrast, regions lacking EVOH passivation undergo oxygen re‐adsorption following UVVH treatment, forming the surface depletion layer and leading to high‐resistivity ZnO (HR ZnO) development.^[^ ^10^, ^32^, ^33^ ^]^ The operational area of the pH sensor is situated at the interface between the LR and HR ZnO NPs. Owing to the porous structure of the nanoparticle film, the pH solution can permeate through the film, allowing it to directly contact the edge of the LR ZnO region, functioning as a liquid electrode. This is confirmed in the I‐V measurement of a device only with carbon electrode and a ZnO NPs device with TiO_x_ and SiO_2_ layer that are exposed to 400 µL of DI water (see Figure 1c). Similar I‐V characteristic is observed in PBS solution (Figure S3, Supporting Information) resistance using the carbon electrode with and without the ZnO, TiOx, and SiO_2_ layers. The I‐V characteristics are due to the water permeating through the nanoparticle film and establishing contact with the electrodes. Figure 1c also shows the cross‐section of the multilayer film, which reveals a porous structure that permits water permeability. Upon immersion in a solution, ZnO NPs facilitate the re‐adsorption of water molecules onto their surface, forming surface hydroxyl groups.^[^ ^34^, ^35^ ^]^ The surface hydroxyl groups interact with ions present in the solution, leading to the generation of an electrical potential.^[^ ^36^ ^]^ The underlying mechanism of surface charging involves the adsorption of protons or hydroxide ions by the hydroxyl functional groups on the oxide surface.^[^ ^9^, ^15^ ^]^ Specific hydrogen ion binding sites on the ZnO surface will be protonated in an acidic environment, establishing a positive charge. In alkaline conditions, the adsorption of hydroxide ions occurred. It leads to the development of a negative charge. The point of zero charge (PZC), defined as the pH at which the net charge on the surface is minimized, is observed at approximately pH 9.^[^ ^36^ ^]^ This means, when the pH value of the solution is less than 9, the ZnO NPs surface predominantly exhibits a positive charge, whereas, at pH value exceeding 9, the surface is primarily characterized by negative charges.

### 2.2 I‐V Characterization of LR‐HR ZnO NPs Interface

**Figure 2:**
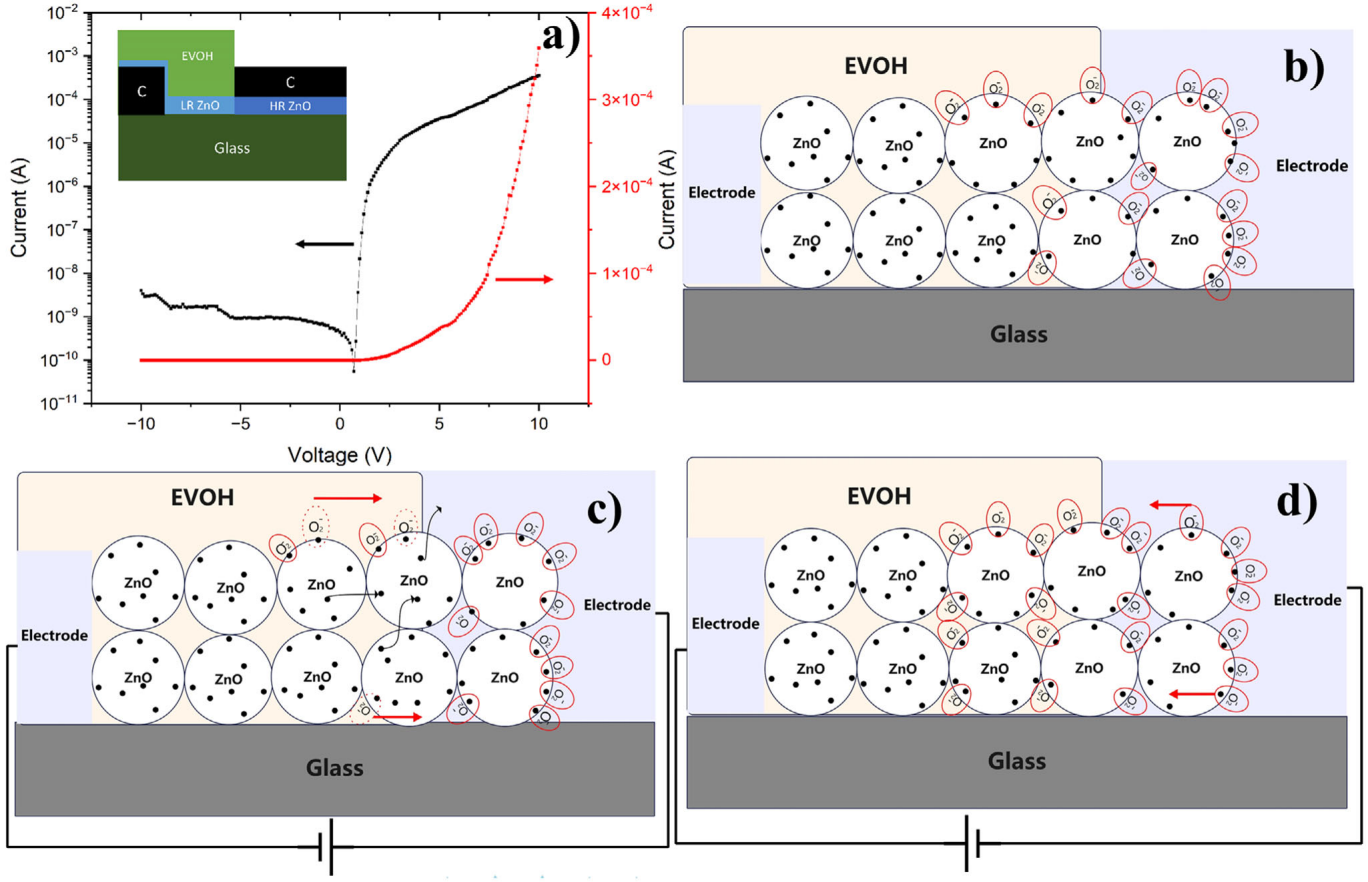
a) The intrinsic diode‐like behavior of the LR‐HR ZnO NPs interface with a solid carbon electrode b) The carrier status without a bias voltage. After UVVH, the ZnO NPs at the LR‐HR interface re‐adsorb the oxygen molecules on its surface. The re‐adsorbed oxygen molecules trap the electron and are negatively charged, forming a surface depletion layer between the electrodes and the LR ZnO NPs film c) With forward bias applied, the negatively charged adsorbed ionized oxygen molecules desorb, out of the LR‐HR ZnO NPs interface, and the depletion layer is reduced. The electrons can freely transfer from the LR ZnO to the electrode, and the device is turned on. d) With the reverse bias applied, the adsorbed ionized oxygen molecules re‐adsorb onto the LR‐HR ZnO NPs interface, increasing the depletion layer between the electrode and the LR ZnO NPs, and the device is turned off.

To demonstrate that the intrinsic properties of the LR‐HR ZnO NPs interface enable characterization in both solid and liquid electrode environments, **Figure** **2**a presents the diode‐like electrical characterization of UVVH‐treated ZnO NPs in a liquid‐free electrode environment. The fabrication process is detailed in Figure S4 (Supporting Information). Following the deposition of ZnO NPs and EVOH with subsequent UVVH treatment, a carbon electrode solution was printed onto the HR ZnO NPs (without EVOH) and dried at room temperature. Voltage was swept from −10 to 10 V, yielding a turn‐off current of 3.9 × 10⁻⁹ A at −10 V and a turn‐on current of 3.5 × 10⁻⁴ A at 10 V, resulting in an on/off ratio of ≈10⁵. The threshold voltage was observed at 4.9 V. Figure S5 (Supporting Information) depicts the I‐V characteristics of the LR ZnO fully covered by the EVOH, exhibiting linear behavior characteristic of working as a resistor. This confirms that the LR‐HR interface is essential for diode‐like behavior.

When studying the effect of surface adsorption on the electrical properties of ZnO NPs, the oxygen molecules adsorbed on the surface of ZnO NPs are a factor that cannot be ignored because the amount of surface adsorption will significantly affect the resistivity of ZnO NPs.^[^ ^10^, ^37^, ^38^, ^39^ ^]^ The following formula can express the relationship between the resistivity of ZnO NPs and the surface‐adsorbed oxygen:^[^ ^10^, ^40^, ^41^ ^]^

(1) $$O_{2}\left(g\right)+e^{-}\to O_{2}^{-}\left(\mathrm{ad}\right)$$

where O_2_(g) represents oxygen molecules in the atmosphere, e^−^ represents the free electron in the ZnO NPs, and $O_{2}^{-}\left(\mathrm{ad}\right)$ represents the ionized surface adsorbed oxygen molecules. In an ambient air environment, oxygen molecules interact with free electrons on the surface of ZnO NPs, leading to ionization. This interaction reduces the free carrier concentration within the ZnO NPs. Additionally, ionized oxygen molecules serve as scattering centers, decreasing the electron mobility in the semiconductor film and consequently increasing its resistivity.^[^ ^42^ ^]^

Figure 2b–d illustrates the proposed mechanism of carrier transport underlying the diode‐like behavior of UVVH‐treated ZnO NPs. Figure 2b depicts the state after UVVH treatment. Due to the ultra‐thin EVOH layer at the LR‐HR interface, ZnO NPs can re‐adsorb oxygen molecules onto their surface. These re‐adsorbed oxygen molecules trap electrons and acquire negative charges, forming a low‐conductivity surface depletion layer between the electrodes and the LR ZnO NPs film. Based on Greenham et. al,^[^ ^31^, ^43^, ^44^ ^]^ applying varying offset voltages to both ends of ZnO NPs influences the adsorption and desorption dynamics of ionized oxygen molecules on their surface. This phenomenon is hypothesized as the result from the migration of positively charged oxygen vacancies within the ZnO NPs under the applied bias voltage. This migration modulates the surface adsorption charges, consequently altering the resistance of the ZnO NPs. As shown in Figure 2c, under a forward bias voltage, the negatively charged adsorbed ionized oxygen molecules desorbed from the LR‐HR ZnO NPs interface. This reduces the surface depletion layer between the ZnO NPs interface and the electrode, facilitating electron transfer from the LR ZnO to the electrode and turning the device on. Figure 2d shows that when a reverse bias is applied, the adsorbed ionized oxygen molecules adsorb onto the LR‐HR ZnO NPs interface, expanding the depletion layer between the interface and the electrode. This enlarged depletion layer inhibits electron transfer, effectively turning the device off. Figure S6 (Supporting Information) shows the hysteresis characterization of the diode‐like device. The hysteresis from the forward and backward sweep of the threshold voltage is 4.7 V. The repeated bias sweep shows a positive threshold voltage shift. This shift is likely due to the electric‐field‐induced movement of ionized oxygen molecules, which exposes additional binding sites to ambient oxygen, and traps more free electrons within the ZnO nanoparticles. Implementing an additional passivation layer at the interface is expected to enhance device stability.

### 2.3 pH Measurement Results and Discussion

**Figure 3:**
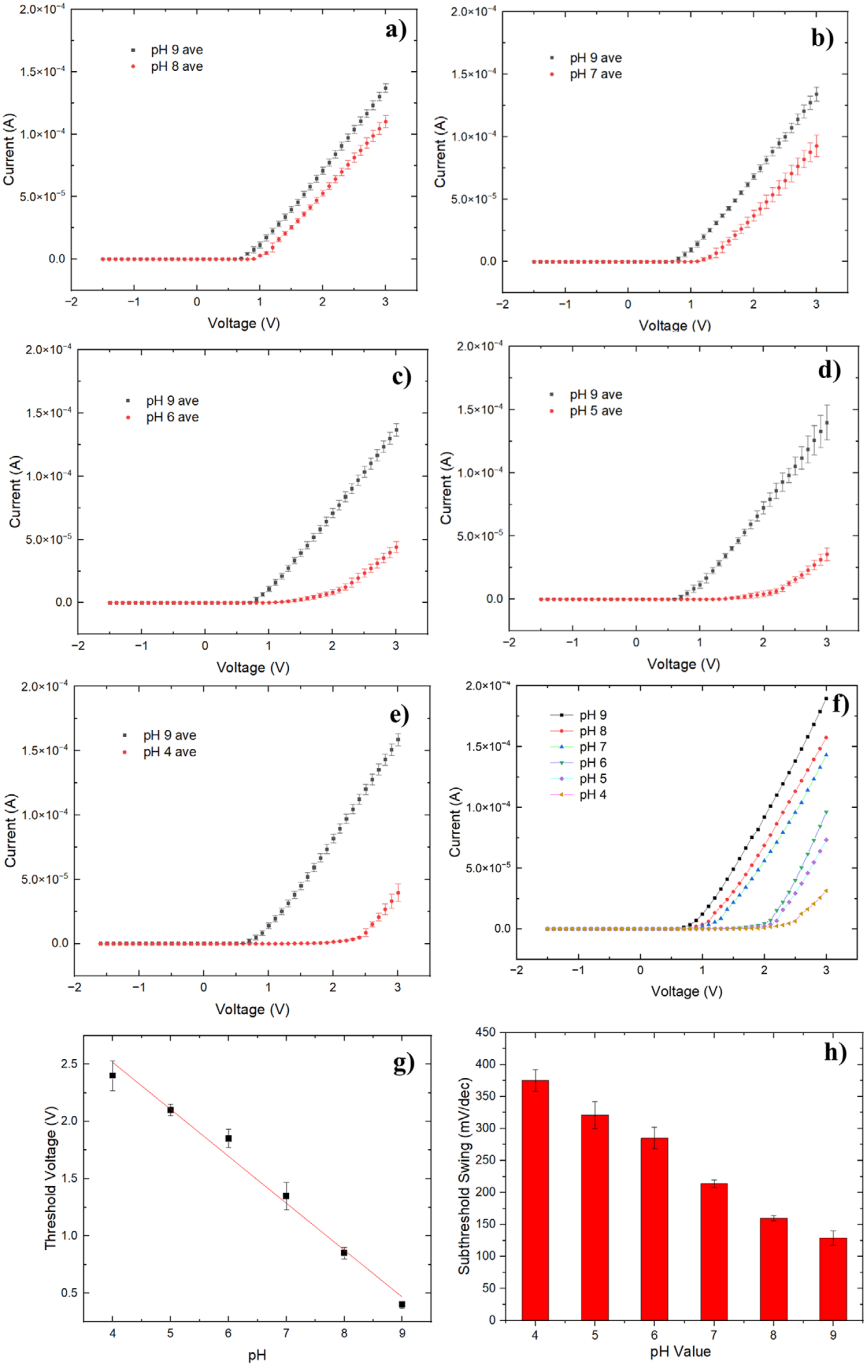
a–e) Diode‐like pH sensor behavior for five devices. They all start with measuring pH 9 as the reference and then the target pH level. The threshold voltage increases as the pH level applied to the device decreases. f) One device measures different pH solutions g) The titration curve shows the sensor sensitivity is 360 ± 11 mV pH^−1^. h) The subthreshold swing of different pH detection.

For the pH measurement, a 50 µL aliquot of the pH solution was applied to the detection region of the pH sensor devices (shown in Figure 1a), and a 5‐min interval was allowed to ensure sufficient adsorption of ions by the ZnO NPs. **Figure** **3** displays the linear‐scale current‐voltage (I‐V) characteristics of the ZnO NPs pH sensor The ZnO NPs devices exhibit diode‐like I‐V characteristics. The voltage sweep was conducted from −1.5 to 3 V in 0.1 V increments. Figure 3a–e depicts individual pH sensing measurements from five distinct devices. Each pH condition was measured three times through a series of titrating and drying process to obtain the average values. According to Fortunato et al on WO_3_ nanoparticle pH sensors,^[^ ^22^ ^]^ pH 9 was employed as the initial measurement point due to the minimal output voltage of this pH value. Therefore, in this work, a similar approach was used with pH 9 as the initial solution for measurement and calibration, followed by subsequent pH values. This choice is attributed to the minimum surface potential observed in ZnO nanoparticles at approximately pH 9. For the pH 9 measurement, the diode‐like devices exhibit an off‐state current on the order of 10⁻⁹ A and a turn‐on current of ≈10⁻⁴ A, achieving an on/off ratio of 10⁵ at 2.5 V. The threshold voltage for detecting pH 9 is consistently 0.31 V across all devices, increasing progressively as the pH level decreases. Figure 3f illustrates pH detection using a single device across various solutions. The threshold voltage transitions from 0.47 to 2.31 V as the pH decreases from 9 to 4, corroborating the pH measurement results across all devices. The current at 2.5 V decreases from 1.1 × 10⁻⁴ A to 6 × 10^−6^ A. Figure 3g illustrates the titration curve of the device, showing a sensitivity of 360 ± 11 mV pH^−1^ unit for the threshold voltage shift. Compared with the cross‐Nernst limit devices,^[^ ^16^, ^29^, ^30^, ^45^ ^]^ whose resolution ranges from 110 to 362 mV pH^−1^, the resolution of the diode‐like ZnO NPs pH sensor shows reasonable and sufficiently competitive sensitivity value. Besides, the threshold voltage of the intrinsic diode device is higher than that of the pH‐sensing device. This discrepancy is attributed to the suboptimal contact between the second carbon electrode, which is deposited after the UVVH process, and the LR‐HR ZnO interface NPs compared to the liquid electrode. Consequently, adsorbed ionized oxygen molecules at the LR‐HR ZnO interface require more energy to migrate, increasing the threshold voltage.

The subthreshold swing is a critical parameter that characterizes the influence of gate voltage on the channel current of a field‐effect transistor. Additionally, it serves as a measurement indicator for diode devices, elucidating the relationship between forward bias voltage and current.^[^ ^46^, ^47^ ^]^ The I‐V characteristic curves, presented on a logarithmic scale in Figure S7 (Supporting Information), illustrate the current variations of the ZnO NPs diode‐like pH sensor within the subthreshold range. Notably, as the pH value of the measured solution decreases, the subthreshold swing of the device correspondingly increases. As summarised in Figure 3h, for pH values ranging from 9 to 4, the observed subthreshold swing values are: 129 ± 5 mV dec^−1^, 160 ± 13 mV dec^−1^, 214 ± 8 mV dec^−1^, 285 ± 15 mV dec^−1^, 321 ± 21 mV dec^−1^, and 375 ± 17 mV dec^−1^. These findings suggest that, at lower pH levels, the effect of voltage on the device channel current diminishes.

**Figure 4:**
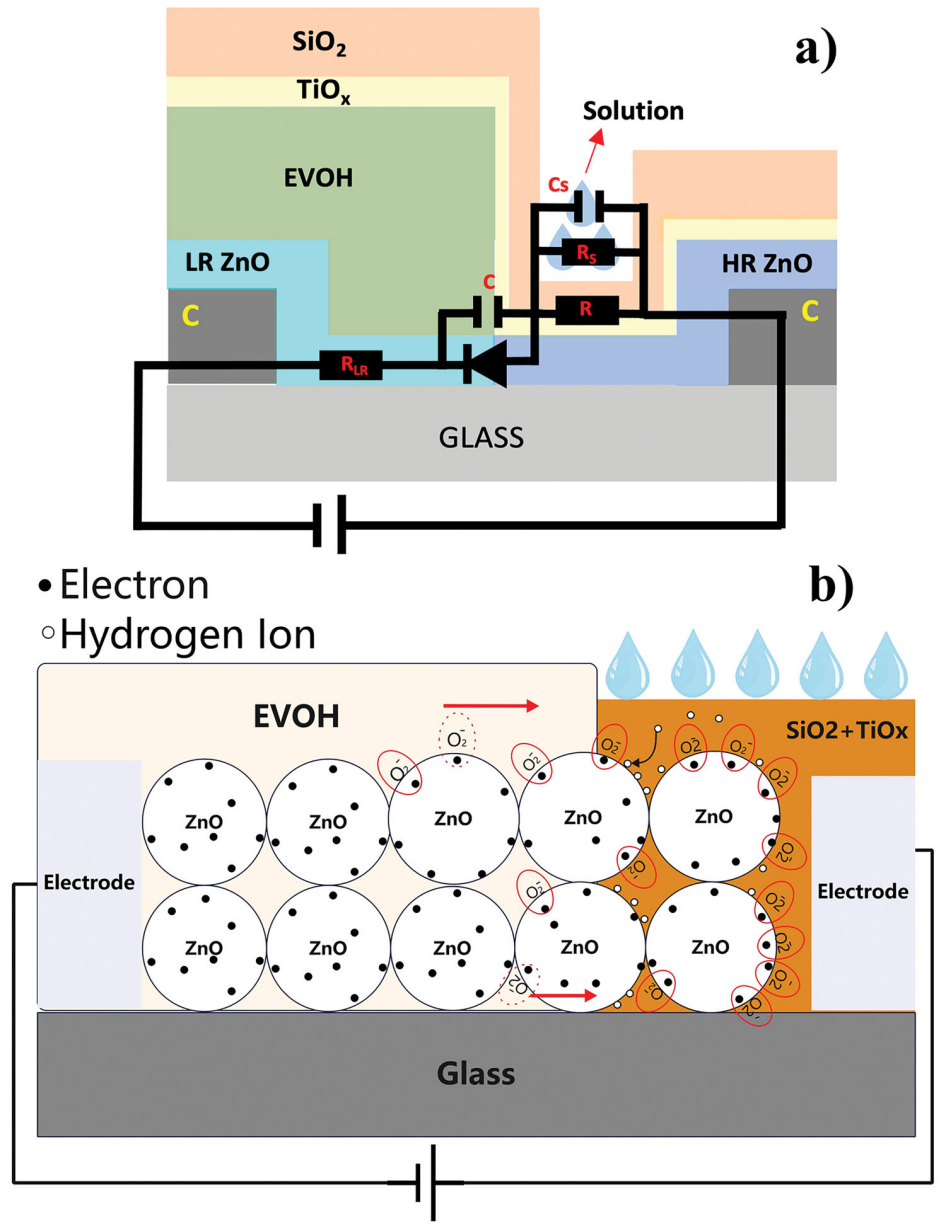
a) The schematic diagram of the ZnO NPs pH sensor structure and the equivalent circuit. R_LR_ and R_S_ represent the resistance of low‐resistance ZnO NPs and the solution, respectively. R represents the resistance of the high resistivity ZnO NPs. The diode represents the interface between the LR ZnO NPs and the HR ZnO NPs. b) With the surface hydrogen ion adsorption, the positive hydrogen ions and adsorbed ionized oxygen molecules form electrostatic forces and attract each other. While applying the forward bias voltage, the ionized oxygen molecules require more energy to migrate, increasing the threshold voltage.

**Figure** **4**a shows the equivalent circuit diagram of the ZnO NPs pH sensor. R_LR_ and R_S_ represent the resistance of LR ZnO NPs and the solution, respectively. R represents the resistance of the HR ZnO NPs. C and Cs represent the capacitance of the LR‐HR interface and the solution. The diode represents the interface between the LR ZnO NPs and the HR ZnO NPs. From this figure, it can be inferred that the current flowing through the sensor under forward voltage bias can be written as:

(2) $$I=V/\left(R_{\mathrm{LR}}+R_{S}+R_{\mathrm{inter}}\right)$$

R_LR_ and R_s_ are fixed values determined through prior measurements of the material resistivity. Previous studies indicate that the contact resistance between the LR ZnO and the electrode is significantly lower than the bulk resistance of the ZnO.^[^ ^10^ ^]^ However, in the HR ZnO, direct contact of the solution with the electrode through permeation could effectively create an interface between the HR ZnO and the electrode. As a result, the contribution of contact resistance is expected to be much lower than the interface resistance stemming from the pH solutions. Therefore, the contact resistance can be combined into the overall low‐resistance region. R_inter_ is the resistance at the LR‐HR ZnO NPs interface, determined by the adsorbed ionized oxygen molecule, which can be affected by the voltage across the sensor and the pH value of the measured solution.

The pH sensing mechanism of ZnO can be attributed to the adsorption of the ions onto the ZnO NPs, forming charged functional groups on its surface.^[^ ^9^, ^15^, ^48^ ^]^ Figure 4b presents the proposed pH‐sensing mechanism. When the pH solution fully penetrates and contacts the HR‐LR ZnO NPs interface, they function as a liquid electrode. Upon adsorption of hydrogen ions from the pH solution, electrostatic interactions form between the positively charged hydrogen ions and the negatively charged adsorbed ionized oxygen molecules. During forward bias application, these interactions impose a higher energy barrier for migrating ionized oxygen molecules, increasing the threshold voltage of the device. A higher hydrogen ion concentration (lower pH value) strengthens this electrostatic force, further elevating the threshold voltage and enhancing the resistivity of the interface.

To elucidate the relationship between the hydrogen ion concentration in the solution and its adsorption on the ZnO NPs, the Freundlich adsorption isotherm was employed as a suitable model to describe multilayer adsorption on heterogeneous surfaces.^[^ ^49^ ^]^ The Freundlich isotherm is expressed as follows:^[^ ^50^ ^]^

(3) $$\frac{X}{m}=K_{f}\times C_{eq}^{1/n}$$

here, x is the adsorbed mass of the adsorbate, m represents the mass of the adsorbent, and C_eq_ represents the adsorbate equivalent concentration in the solution. K_f_ and n are the Freundlich parameters. The Freundlich isotherm describes the relationship between the adsorbate‐to‐adsorbent mass ratio and the equilibrium concentration of the adsorbate. Since the mass of ZnO NPs and the adsorbed hydrogen ions is negligible compared to the pH solution mass, the equilibrium concentration of hydrogen ions is effectively equivalent to the initial solution concentration. The mass of adsorbed hydrogen ions can thus be written as:

(4) $$x_{H}=K_{f}\times C_{eq}^{1/n}\times m_{a-ZnO}$$

x_H_ is the mass of adsorbed hydrogen ions, and m_a‐ZnO_ is the mass of LR ZnO NPs at the interface. PZC of ZnO NPs is approximately at pH 9; when ZnO NPs are in a solution with a pH lower than this value, the surface charge of the nanoparticles becomes positive. The hydrogen ion can interact with the surface compensation of the metal oxide surface. The adsorption and the desorption of the ionized oxygen molecules on the ZnO NPs can be controlled by the voltage field applied to the film.^[^ ^31^, ^43^, ^44^ ^]^ Adsorption of hydrogen ions onto the surface of ZnO NPs induces the attraction of adsorbed ionized oxygen molecules, thereby increasing the energy required for their migration. It has been proposed that the density of the counter ions adsorption site is proportional to the density of the potential‐determining ion on the metal oxide surface, with counter ions compensating for the potential‐determining ions to form ion pairs.^[^ ^15^ ^]^ Consequently, a higher degree of hydrogen ion adsorption leads to an increased amount of remaining adsorbed ionized oxygen molecules under a fixed forward voltage bias. It can then be assumed that the amount of adsorbed ionized oxygen molecules is proportional to the amount of adsorbed hydrogen ions under these conditions.

(5) $$n_{ox}\propto \frac{x_{H}\;}{M_{H}}\;\times N_{A}\;$$

(6) $$n_{ox}=\frac{a\;\times \;K_{f}\times \;C_{eq}^{1/n}\;\times N_{A}\times m_{a-ZnO\;}}{M_{H}}$$

 n_ox_ represents the amount of the remaining adsorbed ionized oxygen molecules. M_H_ represents the molar mass of hydrogen ions. Parameter *a* serves as the fitting constant, balancing the adsorbed oxygen molecules and hydrogen ions. Given the electrostatic nature of the interaction between the hydrogen ions and adsorbed ionized oxygen molecules, the value of *a* is influenced by the forward bias voltage. Ideally, an increase in the forward bias breaks this interaction and reduces the value of *a*. While the presence of adsorbed ionized oxygen molecules is known to increase the resistivity of ZnO NPs films, the precise quantitative impact requires further investigation and validation. An interpretation suggests that adsorbed ionized oxygen molecules enhance carrier scattering within the material, consequently diminishing carrier mobility. Furthermore, carrier mobility is inversely proportional to the areal density of these adsorbed ionized oxygen molecules.^[^ ^42^, ^51^ ^]^ When the carrier concentration is defined, the relationship between carrier mobility and the density of adsorbed ionized oxygen molecules can be articulated as follows:^[^ ^42^ ^]^

(7) $$\frac{1}{\mu_{\left(N_{ox}\right)}}=\frac{N_{ox}}{C_{\left(n\right)}}+\frac{1}{\mu_{0}}=\frac{n_{ox}}{A_{nps}\times C_{\left(n\right)}}+\frac{1}{\mu_{0}}\;$$

here µ_(Nox)_ represents the carrier mobility of adsorbed ZnO NPs. N_ox_ is the areal density of the adsorbed ionized oxygen molecule, representing the carrier mobility at the interface. A_nps_ is the total surface area of the ZnO NPs. µ_0_ corresponds to the pristine mobility of ZnO NPs without adsorption, the same as the LR ZnO NPs. The coefficient C_(n)_ reflects the scattering characteristics resulting from adsorption, which is influenced by carrier concentration and adsorption properties. Given that the resistivity *ρ = 1/n_e_µq*, where n_e_ is the carrier concentration and q is the elementary charge. Resistance *R = ρL_cross_/A_cross_*, where L_cross_ and A_cross_ are the length and cross‐sectional area of the ZnO NPs interface. So, the resistance of the ZnO NPs with adsorbed ionized oxygen molecules can be expressed as:

(8) $$\begin{array}{ccc} R_{inter} & & =\frac{L_{cross}}{A_{cross}\times A_{nps}\times n_{e}\times q\times C_{\left(n\right)}\times M_{H}}\\ & & \times a\times K_{f}\times C_{eq}^{1/n}\times N_{A}\times m_{a-ZnO}+\frac{L_{cross}}{A_{cross}\times n_{e}\times q\times \mu_{0}} \end{array}$$

From Equation (8), the term *L_cross_/(A_cross_ × n_e_ × µ_0_ × q)* can be interpreted as an extension R_LR_ in the LR‐HR interface, allowing it to be incorporated into R_LR_. Extract the coefficient *L_cross_/(A_cross_ × A_nps_ × n_e_ × q × C_(n)_ × M_H_)*, and a coefficient *b = L_cross_ /(A_cross_ × A_nps_ × n_e_ × q × C_(n)_ × M_H_)* is defined. By substituting Equation (8) into Equation (2), the relationship between the forward bias current and the hydrogen ion concentration is established.

(9) $$I=\frac{V}{R_{LR}+R_{s}+a\times b\;\times K_{f}\times C_{eq}^{1/n}\;\times N_{A}\times m_{a-ZnO\;}\;}\;$$

**Figure 5:**
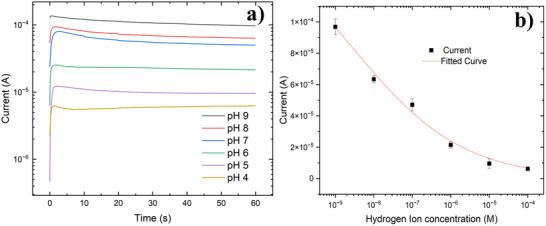
a) The time‐dependent measurement for the ZnO NPs pH sensor shows a stable character. The voltage between the electrodes was set at 2.5 V. b) The current value from a for each pH measurement at 60th s, and the fitted curve is obtained from the derived Equation (9).

**Figure** **5**a depicts the time‐dependent current response of a ZnO NPs‐based pH sensor under an applied voltage of 2.5 V. Measurements were recorded five minutes after introducing the pH solution to the sensor. Figure S8 (Supporting Information) shows the output current of pH 9, 7, and 4 measured over a period of 1‐h. The measurements were done on three individual devices. The results show a reduction in the output current as the pH value decreases, which may be attributed to the adsorption of additional oxygen molecules from water during the measurement or electrophoretic movement of the nanoparticles,^[^ ^52^ ^]^ both of which can increase the resistance at LR‐HR interface. Figure 5b correlates the current at the 60th s with proton concentration, providing further insight into the influence of surface hydrogen ion adsorption on the electrical properties of ZnO NPs devices.

The fitted curve using the above formula is shown in Figure 5b. The sum of R_LR_ and R_s_ is set as 15 KΩ, calculated from the film resistance of the fully passivated film. The forward bias voltage is set at 2.5 V. The coefficient of determination (r^2^) of the fitting curve is 0.98, which proves that the correlation between the fitting curve and the actual data is very high. It is worth mentioning that the fitting curve only considers the effect of ion adsorption on the electrical properties of ZnO NPs and does not consider the impact of ions generated by chemical reactions on the overall current. Therefore, this process is not applicable in very strong acid and base environments, and the fitting function is limited to low‐bias voltage applications because at higher bias voltage there is a risk of electrolysis of the solution.

The fitting coefficient results: n = 3.24 ± 0.16; *a*  × b  × *K_f_* ×  *N_A_*  ×  *m*_*a* − *ZnO*_= 7.04 × 10^6^ ± 2 × 10^5^. Because the thickness of the film measured by SEM is between 1.8 and 2.2 µm and the particle diameter is assumed as 100 nm. Some of the fitting parameters can be estimated. The mass of total ZnO NPs providing adsorption sites is estimated to be between 2 × 10^−8^ and 6 × 10^−8^ g, which is calculated from the volume and the density, and the surface area is estimated to be 3 × 10^−7^ to 7 × 10^−7^ m^2^. From the datasheet of ZnO NPs (100 nm diameter, Merck), the surface area shows a value of 10 to 25 m^2^ g^−1^, which is consistent with the calculation (12 to 35 m^2^ g^−1^). The carrier concentration is estimated to be 2 × 10^17^ to 8 × 10^17^ cm^−3^, and the coefficient C_(n)_ reflecting the scattering characteristics can be estimated between 10^13^ and 10^14^ V^−1^s^−1^. From these estimations, *a*  × *K_f_* can be calculated between 0.5 and 23, representing the adsorption capacities of ZnO NPs for the ionized oxygen molecules. *K_f_* represents the adsorption capacities for the hydrogen ions and *a* represents the relationship between adsorbed hydrogen ions and adsorbed ionized oxygen molecules. From the literature,^[^ ^49^ ^]^ Freundlich adsorption capacities of ZnO NPs range from 0.25 to 264.9. Although these are from different adsorbates, this proves that the adsorption capacity calculated in this paper is within a reasonable range. **Table** **1** compares the sensitivities of various pH sensors, demonstrating that the two‐terminal ZnO NPs device developed in this study is comparable to the state‐of‐the‐art pH sensors.

**Table 1:** Comparison of different pH sensor types, materials, and sensitivity.

Sensor types	Sensor material	Sensitivity [mV pH^−1^]
Field effect transistor^[^ ^53^ ^]^	ZnO	38
Field effect transistor^[^ ^54^ ^]^	ZnO/silicon nanowire	46
Charge‐Coupling device^[^ ^30^ ^]^	SiO_2_/Al_2_O_3_	240
Electrochemical^[^ ^55^ ^]^	Iridium oxide	51.7
Field effect transistor^[^ ^16^ ^]^	WSe_2_/MoS_2_	362
Extended gate sensing^[^ ^56^ ^]^	ZnO nanorod	53.55
**This Work**	**ZnO NPs**	**360 ± 11**

## 3 Conclusion 

This paper presents a ZnO NPs diode‐like device that diverges from traditional PN or Schottky junctions with an on‐off ratio of 10^5^. A novel pH sensor based on this UVVH‐treated solution‐processed ZnO NPs, exhibits super‐Nernstian behavior achieving a sensitivity of 360 ± 11 mV pH^−1^. A plausible working principle for the sensor is proposed, indicating that the threshold voltage increases from 0.47 to 2.31 V as the pH of the solution decreases from 9 to 4. The current formula derived from the adsorption isotherms aligns well with the experimental data. The observed diode characteristics are attributed to the migration of ionized oxygen molecules adsorbed on the surface of ZnO NPs. In addition, the subsequent adsorption of hydrogen ions inhibits this migration, with the reduction directly proportional to the concentration of hydrogen ions in the solution. The simulation results of the circuit diagram closely correlate with the actual measurement outcomes, further validating the proposed principle. Given its high pH sensitivity, this device demonstrates significant potential for applications such as cancer cell detection. Additionally, the solution‐based manufacturing process, coupled with a maximum processing temperature of 120 °C, is highly suited for integration with flexible electronic fabrication processes.

## 4 Materials and Experiment Section

### Materials and Deposition Process

Commercial carbon ink (Myldan Auto Design) was utilized for electrode printing. ZnO NPs (100 nm, Merck) were dissolved in methanol at a 10% weight ratio, and the resulting solution was subjected to sonication for one hour prior to spin‐coating. For the preparation of the EVOH/DMSO solution, 1.5 g of poly(vinyl alcohol‐co‐ethylene) (ethylene 32 mol%, Merck) was dissolved in 10 mL of dimethyl sulfoxide (DMSO, Merck) and stirred at 90 °C.

Commercial deionized (DI) water from TOP‐UP Water was used to prepare the pH solution. The datasheet shows the DI water has a resistivity of 10^5^ Ω.cm and a significant conductivity of 10 µS cm^−1^. The titanium precursor, titanium diisopropoxide bis(acetylacetonate) (Merck), was diluted 20‐fold in isopropanol (IPA) before use. Colloidal silica (30 wt.% suspension in water, Merck) was diluted 100‐fold in DI water. Hydrochloric acid and sodium hydroxide were used to adjust the phosphate‐buffered saline (PBS, 1×) to various pH levels, which were measured using a commercial pH sensor.

Carbon electrodes were dispenser‐printed with dimensions of 2.5 mm in width and 15 mm in length onto a glass substrate. The ZnO NPs solution was spin‐coated onto the substrate at 2000 rpm for 30 s. A layer of EVOH/DMSO (10 mm in width and 15 mm in length) was printed on the ZnO NPs and heated on a hot plate at 80 °C for 1 h. The titania precursor, titanium diisopropoxide bis(acetylacetonate), was spin‐coated on the ZnO NPs at 4000 rpm for 20 s and heated to 90 °C for 10 min to generate titania particle film. Metal acetylacetonate can be used to form metal oxide nanoparticles by heating.^[^ ^57^ ^]^ The colloidal silica solution was then spin‐coated onto the sample at 4000 rpm for 1 min. Then, the samples were heated at 110 °C for 60 min to form the silica particle film.

### Measurement

All the I‐V measurements were taken in darkness. The current‐voltage (IV) characteristics of the sensor were measured using a Keithley 2401 Source Meter Unit (SMU). Each reading was taken five minutes after applying the pH solution to the detection area. Following measurements and evaporation of the pH solution, the sensors were rinsed with deionized water in preparation for subsequent testing. Two types of pH measurements were conducted. Separate measurements were done on 5 different samples for single pH measurement with reference pH. The measurement was repeated three times for each measured pH, and pH 9 was used as the reference and measured. Additionally, all the pH (9 to 4) measurement was also done on a single sensor.

## Conflict of Interest

The authors declare no conflict of interest.

## Supporting information



Supporting Information

## Data Availability

The data that support the findings of this study are openly available in the University of Southampton's repository at https://doi.org/10.5258/SOTON/D3538.
