# Supplementary material for: Solution‐Processed Diode‐Like ZnO Nanoparticle Device with Tunable Threshold Voltage and Super‐Nernstian Ion Sensitivity
Source: Small. 2025 Jun 12;21(32):2504332. doi: 10.1002/smll.202504332 (PMC12366289; doi:10.1002/smll.202504332)
Supplement: Supplementary file 1 — Supporting Information [file SMLL-21-2504332-s001.docx]

Supporting Information

**Solution-Processed Diode-like ZnO Nanoparticle Device With Tunable Threshold Voltage and Super-Nernstian Ion Sensitivity**

Mengyang Qu*, Huanghao Dai, Omesh Kapur, Stephen. P. Beeby and Harold. M. H. Chong

School of Electronics and Computer Science, University of Southampton,

SO17 1BJ, United Kingdom

E-mail: [mq1g19@soton.ac.uk](mailto:mq1g19@soton.ac.uk)


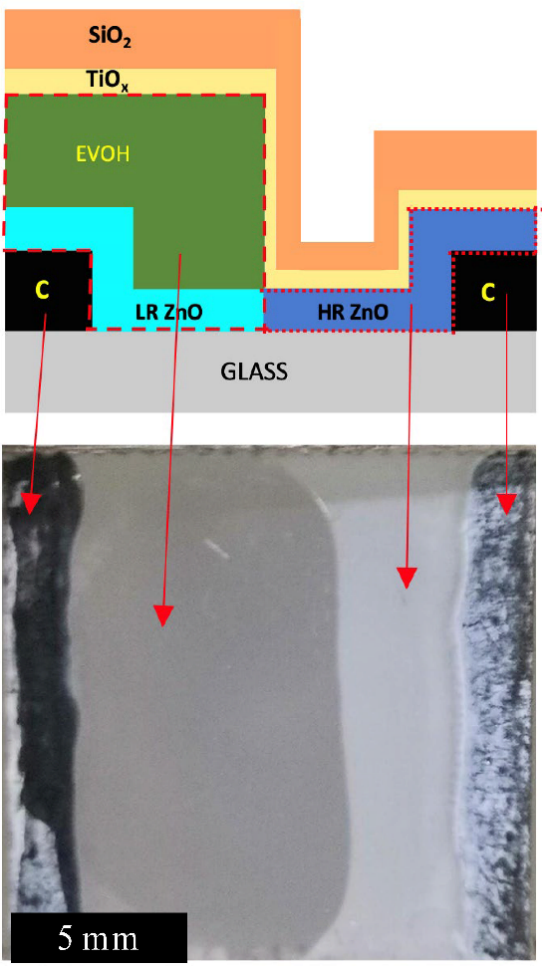


**Figure S1.** UVVH-treated ZnO NPs pH sensors. The length of the carbon electrode is 15mm. The EVOH passivation layer is a 15 mm x 10 mm rectangle.


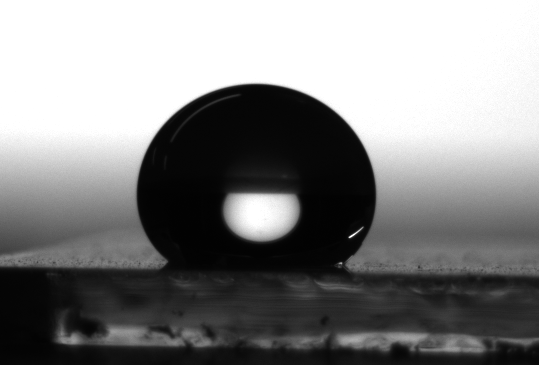

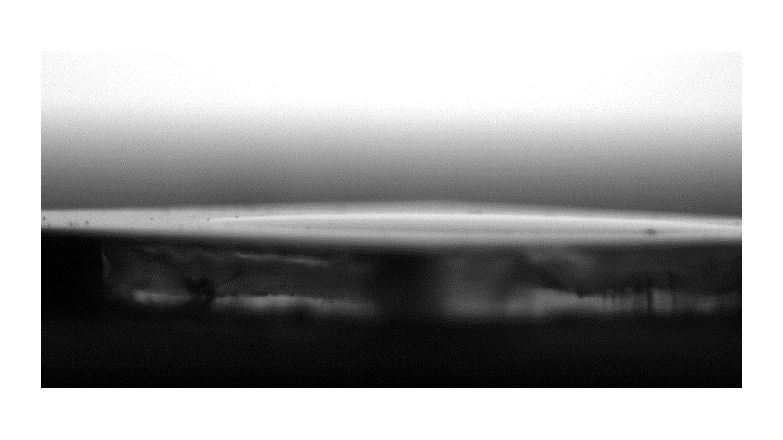

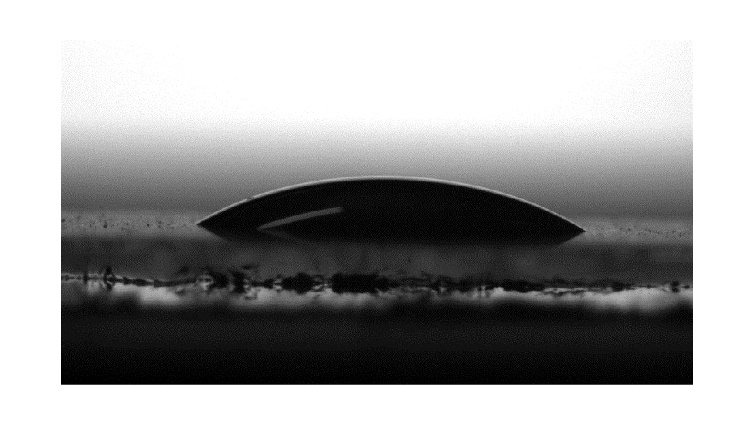


**a)**

**b)**

**c) a**

**Figure S2.** Contact angel measurements for **a)** as deposited ZnO NPs film. **b)** ZnO NPs film after UVVH treatment at 120 ℃. **c)** ZnO NPs film after UVVH treatment at 180 ℃.


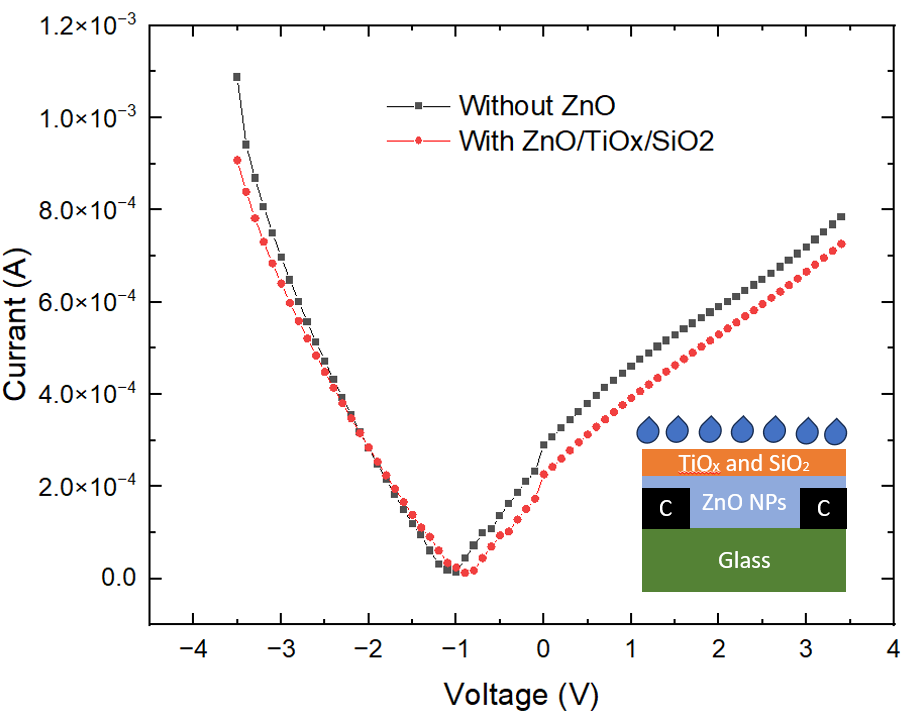


**Figure S3:** I-V curve for the PBS buffer used for pH solution using the carbon with and without the ZnO NPs.


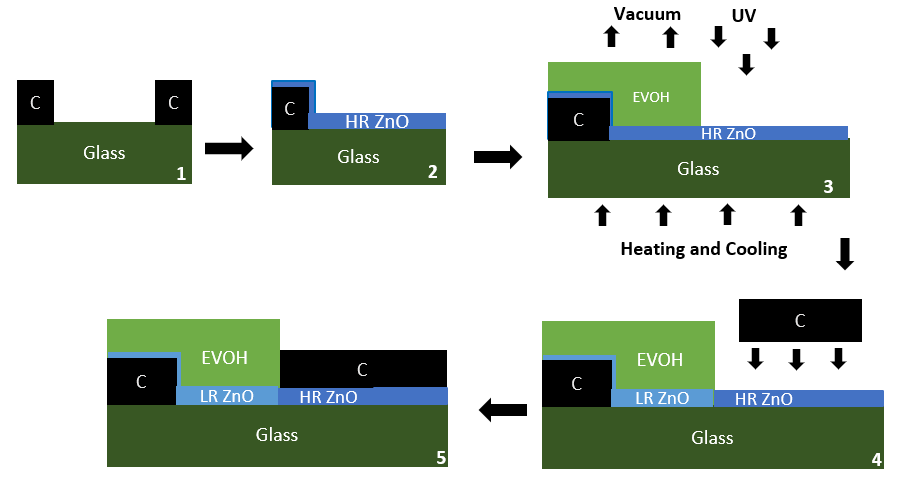


**Figure S4.** Fabrication process of the ZnO NPs diode-like device without liquid electrode.


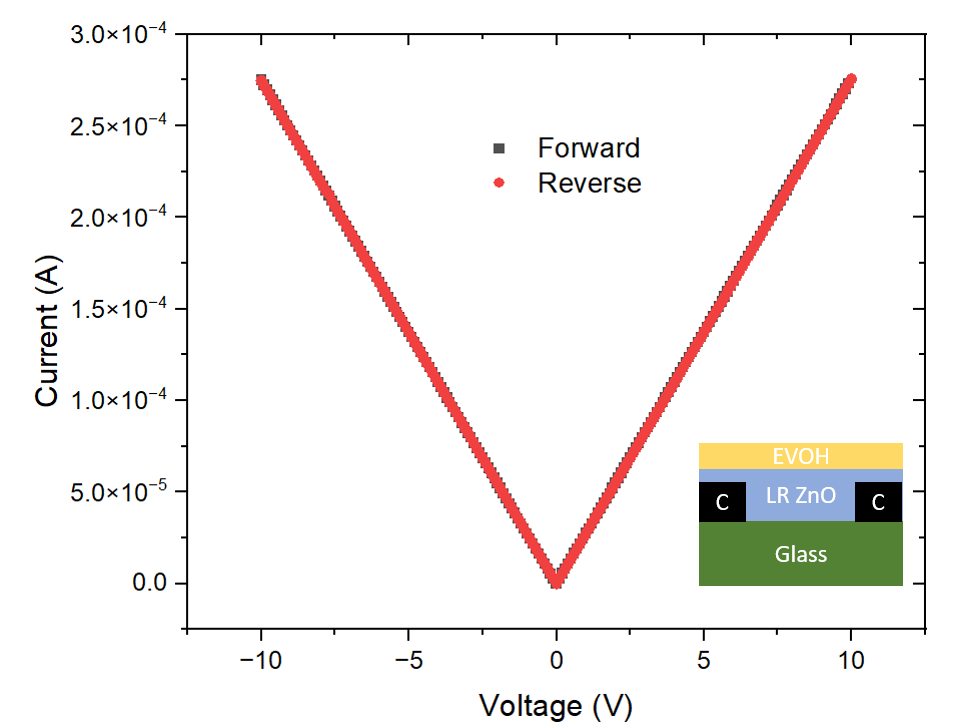


**Figure S5.** I-V measurement of the fully passivated LR ZnO film with EVOH.


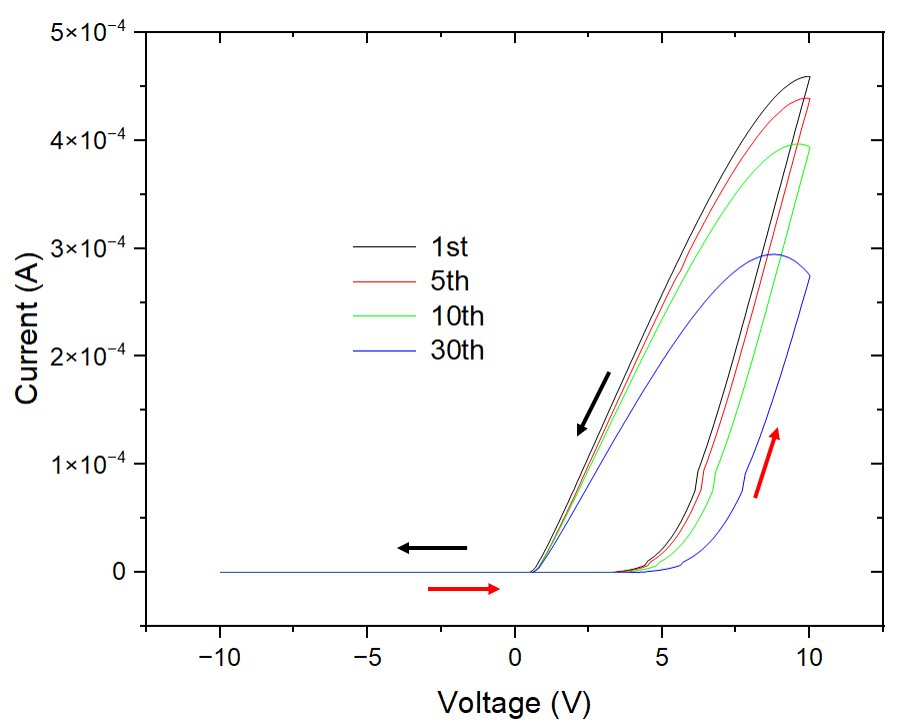


**Figure S6.** Hysteresis measurements for the diode-like ZnO device with dry electrode.


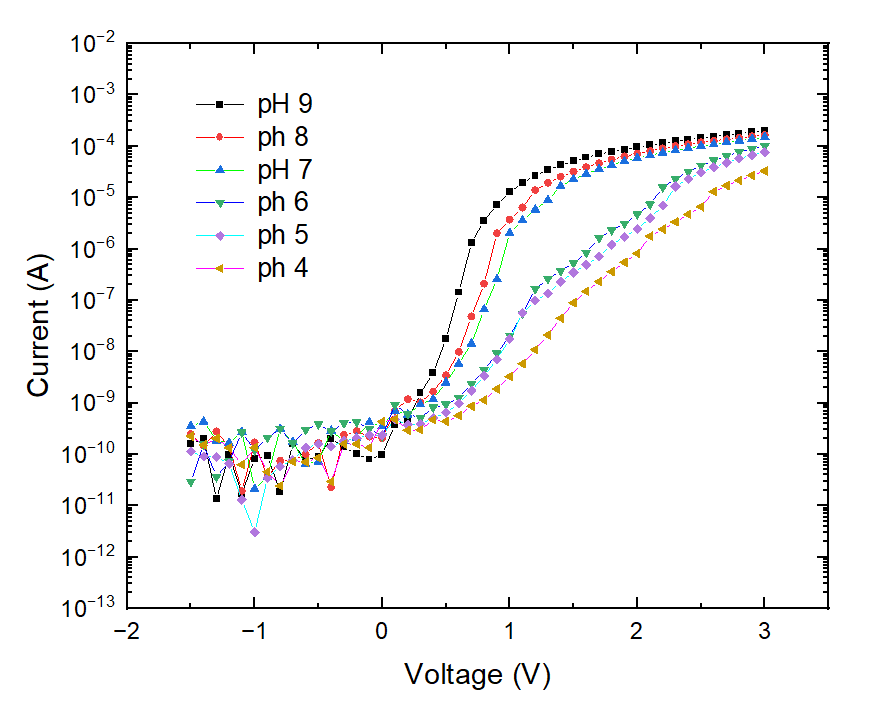

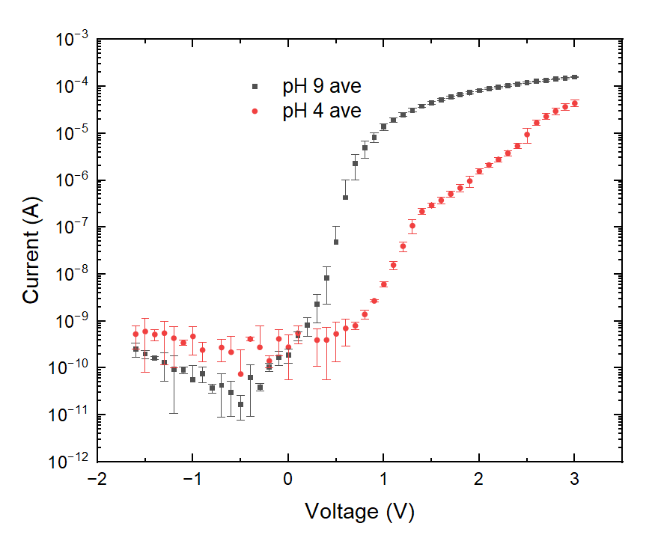

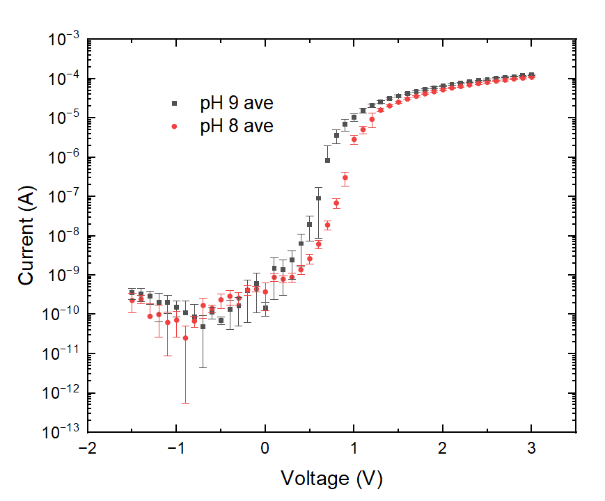

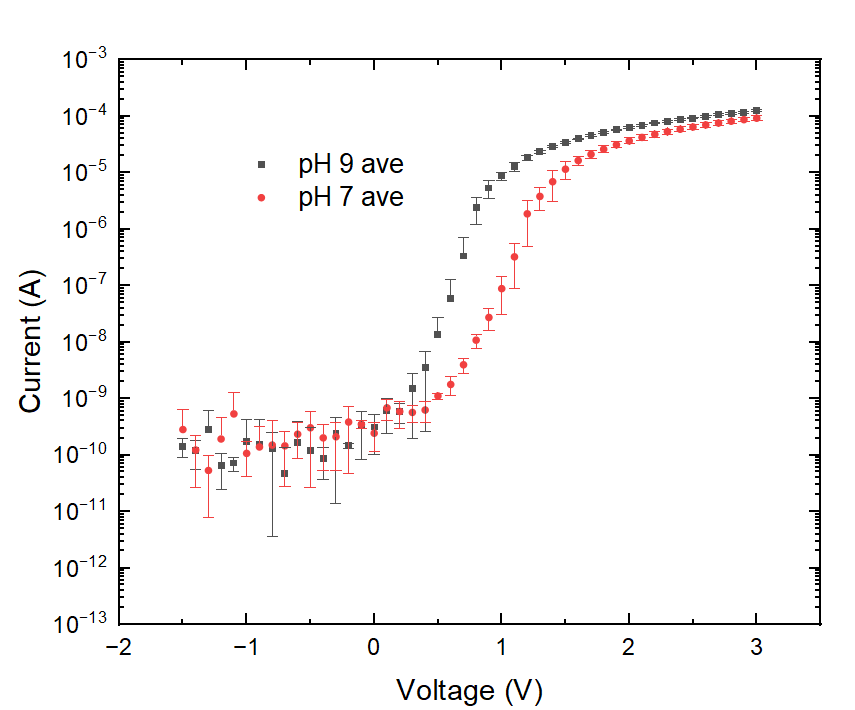

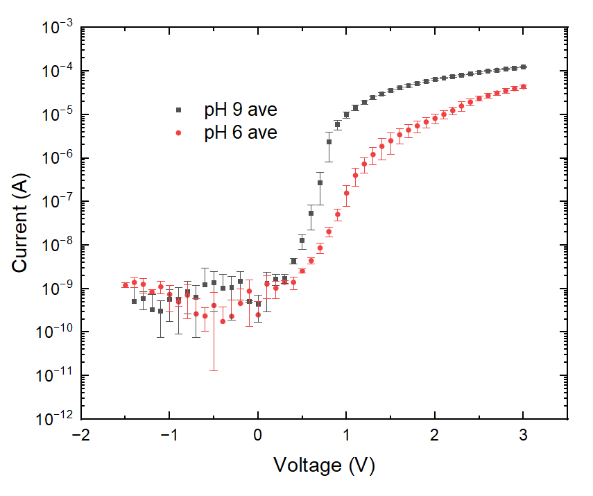

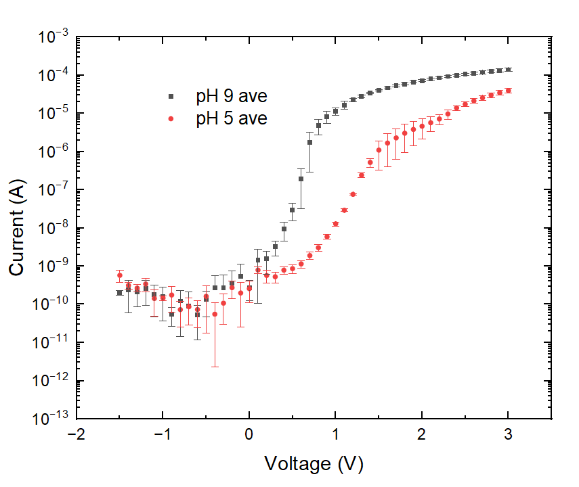


**a)**

**b)**

**c)**

**d)**

**e)**

**f)**

**e)**

**Figure S7. a) - f)** Logarithmic scale IV curve for the ZnO NPs pH sensor.


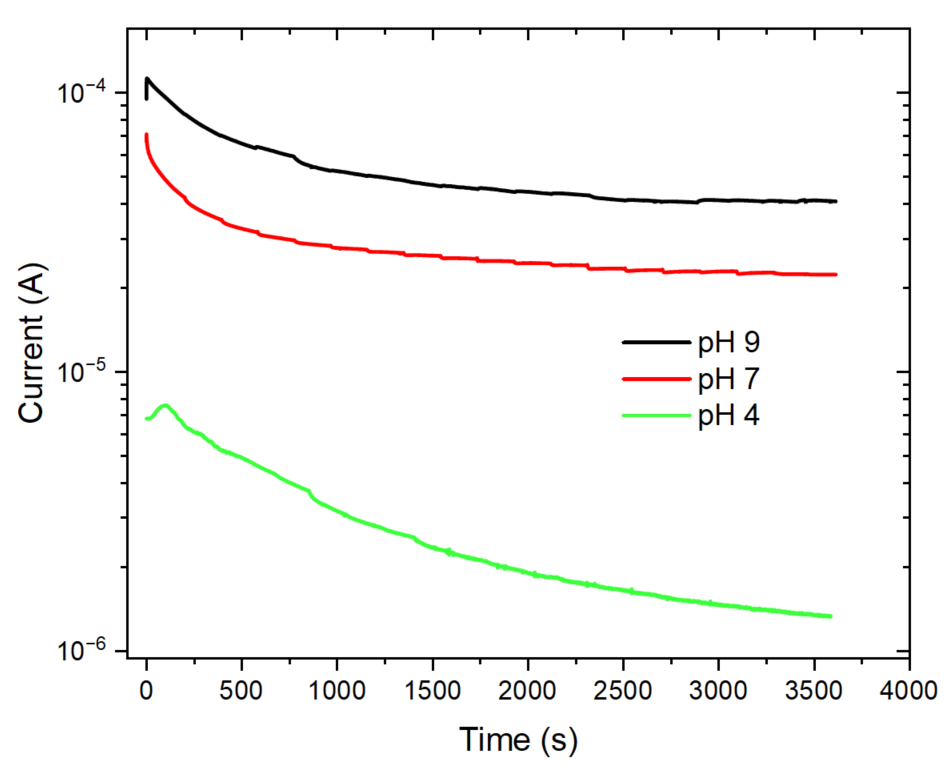


**Figure S8.** One-hour current response for the pH sensor at 2.5 V bias voltage.
